# Functional interaction between immune checkpoints and lipid metabolism in the development of arteriosclerosis obliterans

**DOI:** 10.3389/fimmu.2025.1665454

**Published:** 2025-09-01

**Authors:** Junyi Zhang, Liyuan Cui, Xinhang Meng, Yujie Luo, Jingmin Ou, Songcun Wang, Mingke Qiu

**Affiliations:** ^1^ Department of Interventional Vascular Surgery, Xinhua Hospital, Shanghai JiaoTong University, School of Medicine, Shanghai, China; ^2^ Laboratory for Reproductive Immunology, Obstetrics & Gynecology Hospital of Fudan University, Shanghai, China; ^3^ Shanghai Key Lab of Reproduction and Development, Obstetrics & Gynecology Hospital of Fudan University, Shanghai, China; ^4^ Shanghai Key Lab of Female Reproductive Endocrine Related Diseases, Obstetrics & Gynecology Hospital of Fudan University, Shanghai, China

**Keywords:** arteriosclerosis obliterans, lipid metabolism, immune checkpoints, vascular inflammatory responses, endothelial function

## Abstract

Arteriosclerosis obliterans (ASO) is a chronic vascular disease characterized by narrowing or occlusion of the vascular lumen. Its pathogenesis is complex and closely associated with lipid metabolism disorders and chronic inflammation. Although notable progress has been made in the treatment of ASO, it still remains a cause of surgical limb loss globally. In recent years, immune checkpoints have been identified as critical regulators of the immune microenvironment that play a significant role in ASO. Furthermore, immune checkpoints can affect lipid metabolism by regulating the metabolic pathways of immune cells, thereby indirectly modulating lipid metabolic processes, such as lipid absorption, transport, and degradation, which are crucial in the development and progression of atherosclerosis. Here, we summarized and discussed progress in studies related to lipid metabolism and immune checkpoints during ASO, and highlighted how immune checkpoints regulate lipid metabolism to affect ASO. Further exploration of the interactions between lipid metabolism regulators and immune checkpoints may uncover novel potential therapeutic targets for ASO management.

## 1 Introduction

Arteriosclerosis obliterans (ASO) is a chronic occlusive vascular disease caused by atherosclerosis, mainly affecting the arteries of the lower extremities (1). Due to the progression of atherosclerosis within an arterial lumen, accumulation of atherosclerotic plaques leads to narrowing or even occlusion of the arterial lumen, which further triggers a series of symptoms and signs in the affected limb such as ulcers, gangrene, and even amputation (2, 3). Notable progress has been made in ASO treatment, including surgical techniques, endovascular interventions, and pharmacological treatments. However, restenosis usually relapses within 1 – 2 years after therapy, and ASO remains a cause of surgical limb loss globally (4, 5). Moreover, most patients with early-stage ASOs exhibit no obvious clinical symptoms, leading to delayed treatment. Therefore, early diagnostic markers and new therapeutic approaches for ASO are needed.

Dysregulation of lipid metabolism is a key factor in the pathophysiology of ASO because it promotes lipid deposition, triggers inflammatory responses, and impairs endothelial function. Collectively, these processes drive the development and progression of atherosclerosis (6, 7). Immune checkpoints, the key regulatory molecules of immune activation, can influence plaque formation and vascular function by regulating lipid metabolism and inflammation, and are potentially involved in the occurrence and development of ASO (8–10).

This review summarized the general diagnosis and pathological changes in ASO, and highlighted how immune checkpoints regulate lipid metabolism to cause ASO. By integrating the latest study progress on immune checkpoints and lipid metabolism regulation, novel immune-metabolic combination therapies may be explored to achieve a precise ASO treatment.

## 2 Arteriosclerosis obliterans

ASO is a subtype of peripheral artery disease with increasing global incidence (11, 12). Given its profound impact on patients’ quality of life, early detection, effective prevention, and timely intervention are of paramount importance (13, 14). The diagnosis and treatment of ASO are closely related to atherosclerotic plaques (15, 16). The primary treatment goal is to identify and eliminate arterial plaques, alleviate symptoms, improve quality of life, and reduce the risk of amputation.

### 2.1 Pathology of arteriosclerosis obliterans

Formation of arterial plaques and subsequent vascular narrowing play crucial roles in ASO development (17). The accumulation of atherosclerotic material, coupled with secondary thrombosis and vascular endothelial dysfunction, contributes to the thickening of the intima in lower extremity arteries. This results in the narrowing of arterial lumen and complete occlusion in severe cases (18). These changes lead to a range of clinical manifestations and symptoms in affected limbs. The disruption of lipid metabolism plays an important role in the earliest lesions in ASO (19). The core mechanism involves lipid deposition and chain reactions. Low-density lipoproteins (LDL) penetrate the intima through vascular endothelial cells and undergo local oxidation to form oxidized LDL (ox-LDL). Ox-LDL induces monocytes to adhere to endothelial cells, migrates into the intima, and transforms into macrophages (20, 21). After engulfing ox-LDL, macrophages form foam cells that promote arterial plaques (22). Lipid deposition triggers local inflammatory responses, stimulating the activation of surrounding vascular smooth muscle cells (VSMCs) and fibroblasts (23). Continuous secretion of inflammatory mediators is understood to be a self-amplifying inflammatory cascade that ultimately promotes an unstable plaque phenotype, plaque erosion and rupture, and the formation of occlusive arterial thrombi that restrict blood flow and cause critical tissue ischemia (24–26).

### 2.2 High-risk factors of arteriosclerosis obliterans

Many risk factors, including smoking, age, sex, genetics, diabetes, hypertension, and hyperlipidemia, can lead to ASO, and these factors are often associated with lipid metabolism disorders and inflammation (17, 27).

Smoking is a significant risk factor for vascular diseases, especially those affecting lipids and cytokines, which contribute to vascular damage and ASO. Smokers may have higher concentrations of serum total cholesterol and LDL than non-smokers, increasing their risk of atherosclerosis and coronary artery disease (28, 29). Nicotine and its primary metabolite, cotinine, activate nuclear factor kappa-B (NF-κB) transcription factor, thereby driving tissue factor expression in endothelial cells (ECs) and VSMCs (30).

With aging, vascular walls gradually lose their elasticity and endothelial cell function deteriorates, leading to a reduced capacity for vascular repair following injury (31, 32). Aging is accompanied by chronic inflammation and alterations in lipid metabolism, which further accelerate arteriosclerosis progression (33, 34). Hypertension, hyperlipidemia, and diabetes can also disrupt lipid metabolism and trigger inflammation, all of which are high-risk factors for ASO (35–38). Genetic conditions, such as familial hypercholesterolemia or familial mixed hyperlipidemia, directly affect lipid metabolism pathways, leading to LDL accumulation (39, 40).

Therefore, smoking cessation is critical for the prevention and treatment of ASO. Attention should also be paid to regulating lipid metabolism, such as adopting a healthy diet, controlling blood lipid concentrations, and using lipid-lowering medications (e.g., statins), as part of a comprehensive approach. Moreover, the inflammation triggered by various factors cannot be ignored in ASO.

### 2.3 Lipid metabolism and arteriosclerosis obliterans

Lipid metabolism refers to the entire process of digestion, absorption, transportation, synthesis, breakdown, and utilization of lipid substances in the body (41). Lipid metabolism affects arterial plaques and vascular function through lipid accumulation, fatty acid metabolism, cholesterol transport, and inflammation, thereby contributing to ASO development (42, 43). For example, LDL accumulation in a vessel wall, which is converted into foam cells, and the effect of fatty acids on phenotypic changes in macrophages can exacerbate ASO (44, 45). Additionally, lipid metabolic products, such as oxidized cholesterol derivatives, can activate inflammatory pathways and damage vascular endothelium (46). By activating receptors (such as CD36) on macrophages and VSMCs, ox-LDL triggers an inflammatory response that leads to the progression of atherosclerotic plaques (47, 48). Numerous molecules, such as sterol regulatory element-binding proteins (SREBPs), adenosine monophosphate-activated protein kinase (AMPK), and liver X receptors (LXR) may play pivotal roles in these processes (49–51). Here, we focused on lipid metabolism-related molecules that contribute to ASO.

#### 2.3.1 Sterol regulatory element-binding proteins

SREBPs are a class of transcription factors that play key roles in lipid metabolism, cholesterol synthesis, and fatty acid synthesis (50). SREBP - 1 regulates the transcription of acetyl-CoA carboxylase (*ACC*) and fatty acid synthase (*FASN*) genes to promote lipogenesis, which relies on protein kinase B (Akt)/mammalian target of rapamycin complex 1 (mTORC1) signaling (52, 53). SREBP - 2 overactivation enhance cholesterol synthesis and LDL uptake, leading to hypercholesterolemia (54). SREBP - 2 can directly bind to protein phosphatase 2A or be activated by molecules, such as Erb-B2 receptor tyrosine kinase 4, thereby promoting LDL uptake (55, 56). The SREBP - 2 signaling pathway could be interfered with by histone deacetylase inhibitors (such as butyrate), resulting in a cholesterol-lowering effect (57). Additionally, SREBP - 1c contributes to fatty acid synthesis and lipid accumulation, which exacerbates lipotoxicity and vascular inflammation, and accelerates plaque development (58). In addition to lipid accumulation, cholesterol synthesis also contributes to vascular inflammation, including NOD-like receptor protein 3 inflammasome activation, oxidative stress, and endothelial dysfunction, all of which are key events in atherosclerosis (59, 60). This implies that SREBPs regulate cholesterol metabolism to affect vascular function, further affecting ASO.

#### 2.3.2 Adenosine monophosphate-activated protein kinase

AMPK is a heterotrimeric complex that is activated under conditions of energy stress such as decrease intracellular adenosine triphosphate (ATP) concentrations (61, 62). It reduces lipid accumulation and inflammation by regulating metabolic balance (63). AMPK phosphorylates and inhibits ACC, a key enzyme in fatty acid synthesis that promotes fatty acid oxidation (FAO) (64, 65). Lepropre et al. demonstrated that the AMPK-ACC signaling pathway modulates platelet phospholipid content, thereby regulating arachidonic acid production (62). This process affects thromboxane generation and granule release during platelet activation, ultimately playing a critical role in the regulation of thrombosis formation (66). AMPK also promotes FAO by relieving the inhibitory effect on CPT1A (67). Moreover, AMPK plays a pivotal role in regulating cholesterol concentrations by upregulating ATP-binding cassette (ABC) transporters (e.g., ABCA1 and ABCG1), downregulating the expression of cholesterol synthesis gene 3-hydroxy-3-methylglutaryl-coenzyme A reductase (*HMGCR*), inhibiting SREBP - 1, suppressing the mTORC1 pathway, and mitigating plaque formation (68–71). AMPK activation not only modulates lipid metabolism to mitigate arterial plaque formation but also effectively reduces vascular inflammation. Studies have shown that AMPK activation can suppress the release of pro-inflammatory cytokines, such as interleukin-6 (IL - 6) and tumor necrosis factor-α (TNF-α), which contribute significantly to plaque instability (72, 73). Thus, AMPK reduces foam cell formation, plaque instability, and vascular inflammation, making it a promising therapeutic target for ASO.

#### 2.3.3 Peroxisome proliferator-activated receptors

PPARs are ligand-activated transcription factors belonging to the nuclear receptor superfamily. They regulate the transcription of target genes by forming dimers and binding to specific DNA regions, thereby participating in various physiological processes. There are three main subtypes of PPARs: PPAR-α, PPAR-γ, and PPAR-δ/β (74).

PPARα’s central function in FAO is to regulate downstream genes, promoting the uptake and activation of long-chain fatty acids (75). Activated PPAR-α bound to the retinoid X receptor to form a heterodimer, which initiates the transcription of target genes such as fatty acid transport (FAT) and carnitine palmitoyltransferase-1 (CPT*-*1), thereby reducing lipid accumulation (67, 76). Moreover, PPARα could upregulate lipoprotein lipase and inhibit apolipoprotein C-III expression, thereby reducing triglyceride concentrations in blood (67, 77). In addition to breaking down triglycerides, PPAR-α agonist like LY518674 might promote high-density lipoprotein (HDL) production and reverse cholesterol transport, effectively clearing cholesterol from the vascular walls (78–80). Cholesterol efflux is promoted by the activation of ABCA1 and scavenger receptor class B type I (SR-BI). Notably, a study revealed that SR-BI regulates transcription factor EB expression by enhancing PPAR-α activation (81). This finding identifies SR-BI as a potential new therapeutic target for atherosclerosis (81).

PPAR-γ depends on phosphatidylinositol-3-kinase (PI3K)/Akt/mTOR signal regulation as a key regulator of adipocyte differentiation (82). It promotes the uptake and storage of free fatty acids in adipocytes by inducing the expression of genes, such as fatty acid-binding protein 4 (*FABP4*), thereby reducing the concentration of fatty acids in the bloodstream (83). PPAR-γ upregulates the expression of antioxidant-related genes, such as glutathione peroxidase (*GPx*) and superoxide dismutase (*SOD*), thereby reducing ox-LDL production (84). PPAR-γ might reduce vascular endothelial damage through a reduction of oxidative stress responses (85). At the same time, PPAR-γ decreases ox-LDL uptake by macrophages in vascular walls (86).

PPAR-δ/β activates key downstream genes, such as *CPT-1* and *acyl-CoA oxidase 1* through ligand binding, thereby promoting β-oxidation (87). Additionally, PPAR-δ/β regulates the expression of *FASN* and *FABP*, which were involved in fat synthesis and storage, thereby reducing fat accumulation (67, 88). Furthermore, PPAR-δ/β facilitates lipolysis by activating genes such as *adipose triglyceride lipase (* 89).

PPARs not only regulates lipid metabolism but also play a crucial role in inhibiting inflammation. PPAR-α suppresses the transcription of inflammatory genes by interfering with the NF-κB signaling pathway (90). It also downregulates chemokines and intercellular adhesion molecule-1, thereby reducing monocyte infiltration into the arterial wall (91). PPAR-γ regulates macrophage phenotypes, thereby promoting transition from pro-inflammatory M1 to anti-inflammatory M2 (92). PPAR-δ/β might inhibit the expression of chemokines, such as chemokine ligand 2 and CXC-chemokine ligand-8, reducing the accumulation of inflammatory cells in local tissues (93, 94). In contrast, PPAR-δ/β activates antioxidant genes like *heme oxygenase-1* and *quinone oxidoreductase-1*, enhancing cellular antioxidant defense (95). Furthermore, PPAR-δ/β modulates the production of pro-inflammatory factors (such as vascular cell adhesion molecule-1) in ECs, thereby alleviating inflammatory response in a vascular wall (96). Thus, PPARs have broad application prospects in the prevention and treatment of ASO, as they regulate lipid metabolism, reduce inflammatory responses, and improve vascular function.

#### 2.3.4 Liver X receptors

LXR is a nuclear receptor transcription factor that exists as two main subtypes: LXRα and LXRβ (51). It serves as a critical regulator of lipid metabolism, cholesterol transport, and anti-inflammatory responses by modulating target gene expression. LXR promotes the efflux of cholesterol from macrophages and foam cells to HDL by activating the downstream genes *ABCA1* and *ABCG1*, thereby reducing intracellular cholesterol accumulation (97). LXRα can be upregulated by PPARγ to promote ABCA1 expression and enhance cholesterol efflux (97). Kim et al. found that LXR activation can inhibit toll-like receptor signaling and reduce the expression of inflammatory genes by inducing changes in membrane lipid composition mediated by ABCA1 (98). Additionally, LXR could activate the cholesterol 7 alpha-hydroxylase gene, thereby promoting cholesterol conversion to bile acids and accelerating cholesterol clearance (99). Moreover, LXR plays a role in upregulating SREBP - 1c expression, interaction with AMPK, and collaboration with PPAR-γ to regulate lipid metabolism, further contributing to lipid regulation (100). Therefore, as a crucial factor in lipid metabolism that influences ASO, in-depth studies on the bidirectional regulatory effects of LXR on lipid metabolism and inflammatory responses is essential.

## 3 Immune checkpoints and arteriosclerosis obliterans

**Figure 1:**
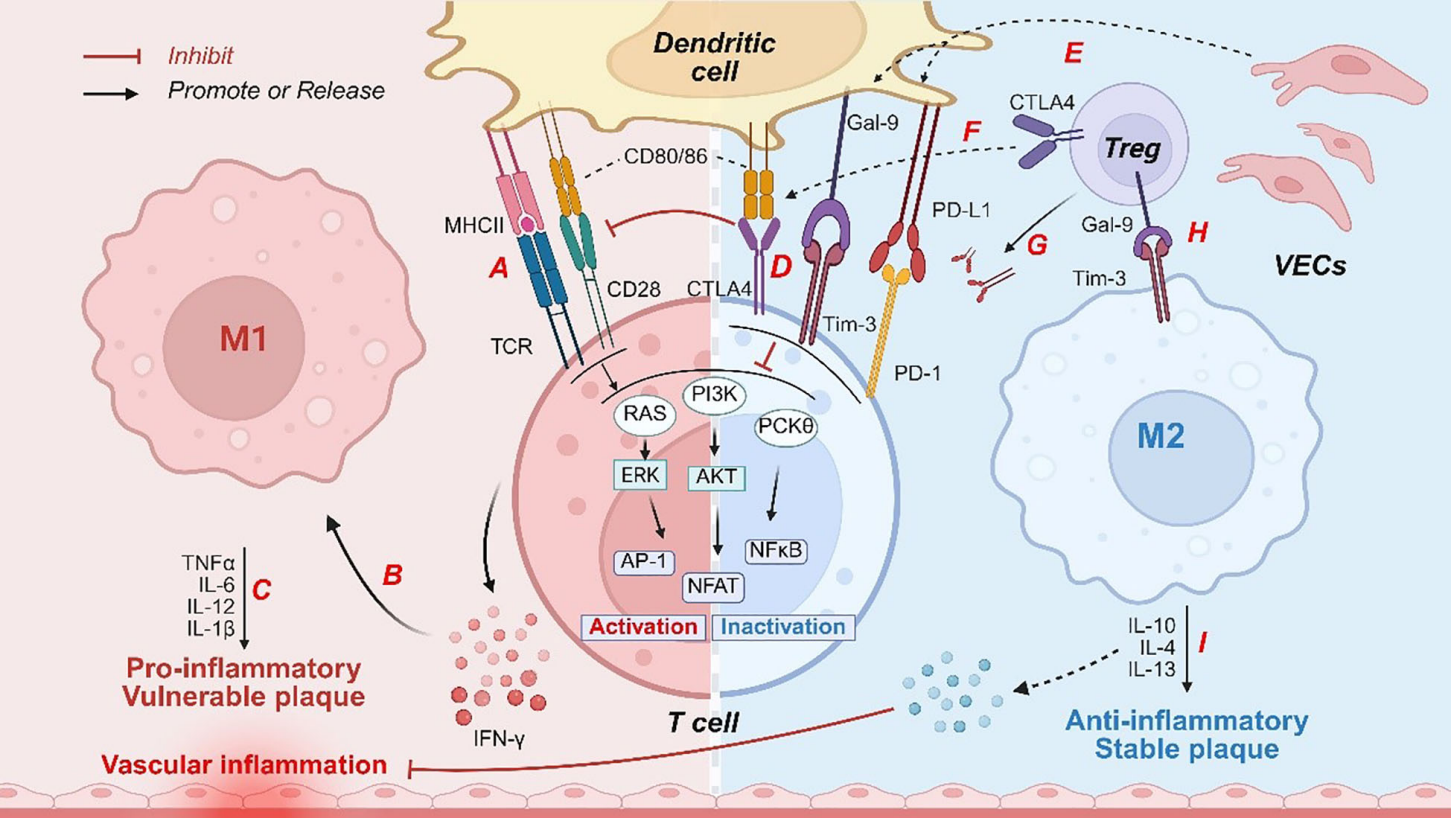
Immune checkpoints regulate immune responses to influence vascular inflammation and plaque stability. Pathogenic factors, such as ox-LDL, induce activation of DCs, characterized by upregulation of **(A)** MHC II molecules and costimulatory markers (CD80/CD86). These activated dendritic cells engage T-cell receptors, initiating downstream signaling pathways including RAS, PI3K, and PKCθ. Subsequently, **(B)** IFN-γ secretion by T cells promotes M1 macrophage polarization. M1-derived **(C)** pro-inflammatory cytokines directly compromise vascular endothelial integrity, exacerbating vascular inflammation and destabilizing plaques. Under inflammatory conditions, **(D, E)** DCs or VECs express ligands for immune checkpoint molecules, such as PD-L1 and Gal-9, which bind to immune checkpoint molecules on T cells (with CTLA4 competitively inhibiting CD80/CD86 binding to CD28), thereby suppressing the signaling pathways associated with T cell activation. Moreover, **(F, G)** CTLA - 4 expressed on Tregs depleted CD80/CD86 and released free PD-L1. **(H)** The Gal-9 secreted by Tregs interacts with Tim-3 receptors on macrophages, driving the polarization of macrophages toward the M2 phenotype. Subsequently, **(I)** M2 macrophages secrete anti-inflammatory mediators that inhibit inflammation and enhance the stability of atherosclerotic plaques. PI3K, phosphatidylinositol-3-kinase; DCs, dendritic cells; PD - 1, programmed cell death protein -1; CTLA - 4, cytotoxic T lymphocyte associated protein 4; Tim-3, T cell immunoglobulin and mucin-domain containing-3; MHC, major histocompatibility complex; TCR, T-cell receptor; Tregs, regulatory T cells; PD-L1, programmed death ligand-1; Gal-9, galectin-9; TGF-β, transforming growth factor β; ox-LDL, oxidized LDL; NF-κB, nuclear factor kappa-B; ECs, endothelial cells; RAS, rat sarcoma; Akt, protein kinase B; AP - 1, activator protein 1; ERK, extracellular signal-regulated kinase; NFAT, nuclear factor of activated T cells; TNF-α, tumor necrosis factor-α; IFN-γ, interferon γ; IL, interleukin.

Immune checkpoints are molecules that regulate immune responses. In most cases, they prevent immune system overactivation, thereby protecting normal cells and healthy tissues from harm (101). Common immune checkpoints include programmed cell death protein 1 (PD - 1), cytotoxic T lymphocyte-associated protein 4 (CTLA - 4), T cell immunoglobulin and mucin-domain containing-3 (Tim-3), etc. These checkpoints play various roles in immune cell activation, differentiation, and immune tolerance (102, 103). Atherosclerosis is closely associated with immune system dysregulation, particularly during endothelial injury, inflammation, and immune cell infiltration (104, 105). Inflammation serves as a core driver of atherosclerosis, connecting traditional risk factors (such as LDL and hypertension) with alterations in vascular wall biology (106). The immune system promotes plaque formation by initiating an inflammatory response (107). By modulating immune cell function and either promoting or inhibiting anti-inflammatory responses, immune checkpoints may influence the critical stages of atherosclerosis, thus playing vital roles in ASO development (**Figure 1**).

### 3.1 Immune checkpoints and immune responses in arteriosclerosis obliterans

After macrophages and dendritic cells (DCs) phagocytose ox-LDL, antigens are presented via major histocompatibility complex (MHC) molecules on the surfaces of antigen-presenting cells (APCs). Subsequently, MHC binding to T-cell receptor (TCR) activates signaling pathways such as rat sarcoma, PI3K, and PKCθ, thereby initiating T cell activation (108–110). However, full activation of T cells requires co-stimulatory signals such as the interaction between CD28 on the T cell surface and CD80/CD86 ligands on the surface of APCs (111).

CTLA-4 is a structural homolog of CD28 and is primarily expressed in activated T cells and regulatory T cells (Tregs). CTLA - 4 binds to CD80/CD86 on the APC surface with high affinity, thereby directly competitively blocking CD28 signaling and inhibiting TCR signal transduction (112). Studies have shown that the overexpression of CTLA - 4 significantly reduces the area of atherosclerotic lesions and decreases the infiltration of macrophages and CD4^+^ T cells within plaques via mechanisms involving the inhibition of CD4^+^ T cell proliferation, downregulation of CD80/CD86 expression, and suppression of T cell activation (113). In contrast, CTLA - 4 inhibition promotes CD4^+^ T cell differentiation to T-helper type 1 (Th1) cells, ultimately exacerbating atherosclerosis (114). Furthermore, CTLA - 4 is a key molecule through which Tregs exert their immunosuppressive functions (115). CTLA - 4 expression on Tregs enhances their immunosuppressive effects by inhibiting the activation of effector T cells and reducing inflammatory responses (116). Tekguc et al. found that Treg-expressing CTLA - 4 depleted CD80/CD86 and released free programmed death ligand-1(PD-L1) on APCs, exerting dual suppressive effects on T-cell immune responses (117).

PD-1 is an inhibitory co-receptor broadly expressed on the surface of activated T cells. Upon binding to its ligand PD-L1/PD-L2, PD - 1 delivers a negative regulatory signal to the cell. In the atherosclerotic environment, PD - 1 suppresses excessive T cell activation and limited Th1 differentiation, thereby reducing the release of pro-inflammatory cytokines such as interferon γ (IFN-γ) and TNF-α (9). A study revealed that in PD - 1 agonist-treated mice, atherogenic IFN-γ-producing splenic CD4^+^T cells and cytotoxic CD8^+^T cells were reduced, while atheroprotective IL - 10-producing CD4^+^T cells were increased. Additionally, the levels of regulatory B cells, B1 cells, and atheroprotective circulating ox-LDL-specific IgM were significantly elevated (118). In PD - 1 and LDL receptor-deficient mice, predominant activation of pro-inflammatory T cells leads to dyslipidemia, vascular inflammation, and atherosclerosis (119).

Tim-3 is an inhibitory receptor expressed on activated T cells, Tregs, macrophages, and DCs, and its primary ligand is galectin-9 (Gal-9) (120). Tim-3 signaling directly induces the apoptosis of pro-inflammatory T cells (such as Th1 cells) (121). It can also regulate inflammatory response by inhibiting NF-κB activation (122). Therefore, the Tim-3 pathway suppresses immune inflammatory responses and exerts atheroprotective effects. Animal experiments demonstrated that administration of anti-Tim-3 antibodies significantly increased the area of lipid streaks and mature plaques, accompanied by an increase in macrophages and CD4^+^ T cells, while the proportion of Treg cells decrease (123). Moreover, our study group found that, at an early stage of atherosclerosis, the proportion of PD - 1^+^ Tim-3^+^ CD8^+^ T cells increased in peripheral or arterial blood of patients. Dual blockade of these two immune checkpoints had led to elevated TNF-α and IFN-γ levels, along with decreased IL - 10 and IL - 4 levels (124). At the higher stage of atherosclerosis, the proportion of PD - 1^+^ Tim-3^+^ CD4^+^ T cells was higher in peripheral or arterial blood of patients. Furthermore, simultaneous blockade of the Tim-3 and PD - 1 signaling pathways exacerbates the pro-atherogenic Th1 response in lower extremity ASO (125). Moreover, Tim-3 signaling drives macrophages toward an anti-inflammatory phenotype. In a glioma study, Gal-9 was shown to activate Tim-3 and its downstream pathways to promote M2 macrophage polarization. Enhanced Tim-3 expression predicts poor prognosis in patients with cancer Conversely, blocking Tim-3 signaling inhibits M2 polarization of macrophages and suppresses tumor growth (126). In the transforming growth factor β (TGF-β)-activated tumor microenvironment, Tim-3 expression was significantly correlated with M2 macrophage polarization. *In vitro* experiments confirmed that TGF-β induced Tim-3 expression in monocytes and M2 macrophages (127). In the context of atherosclerosis, the relationship between Tim-3 and macrophage polarization remains unknown. However, the specific mechanisms by which Tim-3 regulates macrophage inflammatory responses and plaque formation require further exploration.

PD-L1 is expressed in ECs and detected in atherosclerotic plaques. Blocking PD-L1 signaling resulted in a marked increase in IFN-γ^+^ CD8^+^ T cells within plaques, promoting inflammation and worsening atherosclerotic burden (128). Additionally, ECs produce and secrete Gal-9, and plasma Gal-9 levels are elevated in patients with peripheral arterial disease. However, in high-fat diet–fed mice, genetic deletion of Gal-9 led to a significant increase in atherosclerotic plaque formation (129). Although the expression of these EC-derived ligands is upregulated in atherosclerosis, their participation in protecting endothelial cell function and the specific mechanisms remain to be elucidated.

Notably, an increasing number of studies have shown that immune checkpoint inhibitors can precipitate atherosclerotic cardiovascular events (130, 131). For example, preclinical studies have suggested that immune checkpoint inhibitors may exacerbate inflammatory responses in atherosclerosis and promote plaque progression, whereas retrospective studies have further confirmed that immune checkpoint inhibitors could increase the risk of atherosclerotic vascular events (132). Clinical and imaging studies have shown that treatment with immune checkpoint inhibitors is associated with an increased risk of atherosclerotic cardiovascular disease (133). This further confirms the protective role of checkpoint molecules in atherosclerosis and suggests that close attention should be paid to ASO development when using these inhibitors.

## 4 Immune checkpoints in lipid metabolism regulation during arteriosclerosis obliterans

**Figure 2:**
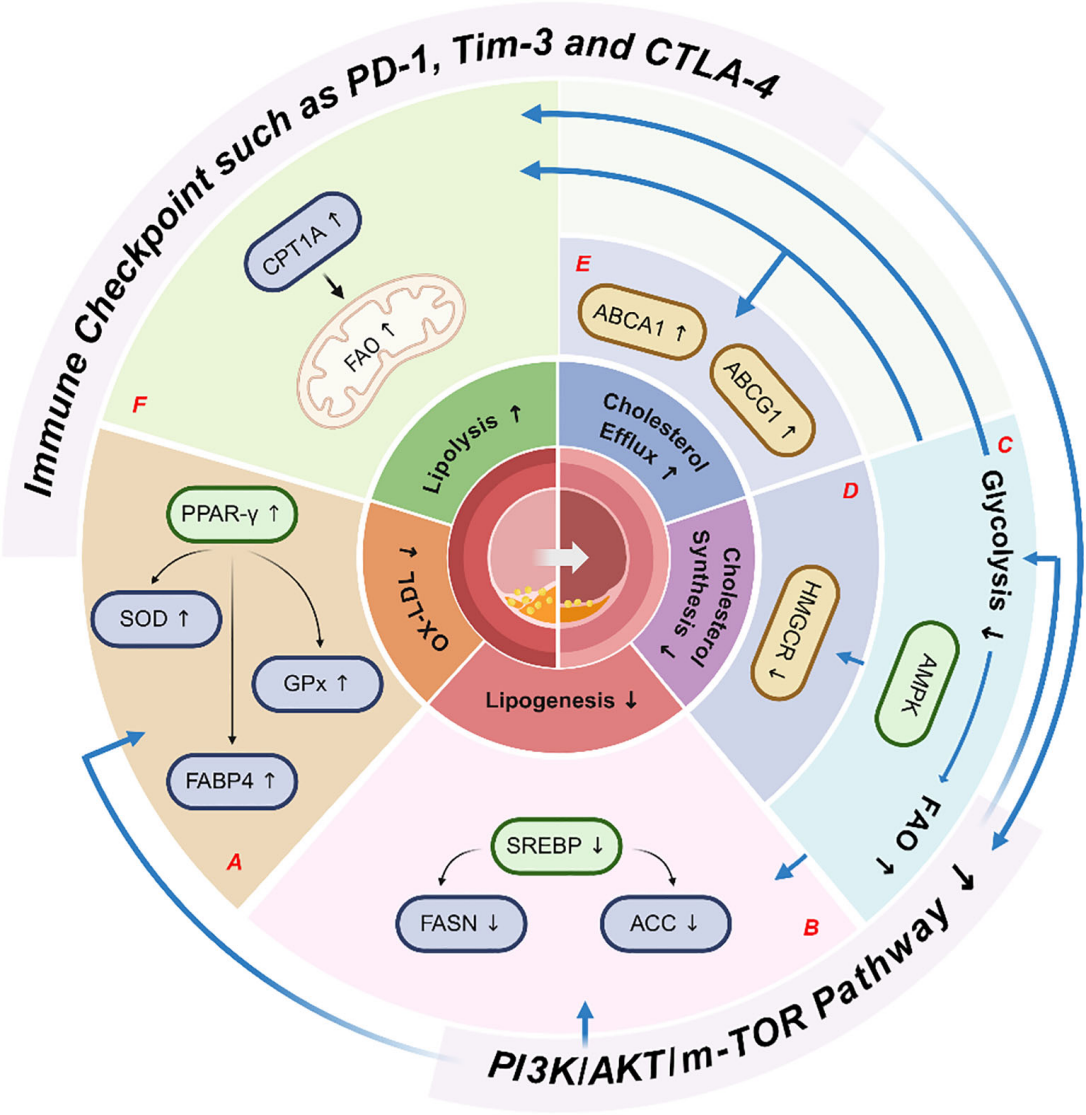
Immune checkpoints regulate lipid metabolism to cause ASO. After binding to its ligand, immune checkpoint molecules cause downregulation of the PI3K/Akt/mTOR signaling pathway, **(A)** thereby upregulating of PPAR-γ and its downstream gene like SOD, leads to a reduction in ox-LDL concentrations, **(B)** leading to a reduction in SREBP concentrations and subsequent downregulation of its downstream target genes, such as FASN and ACC, ultimately resulting in decreased lipogenesis. Following the inhibition of the PI3K/Akt/mTOR signaling pathway, **(C)** the metabolic profile shifts toward the predominant FAO and activates the AMPK pathway. Once activated, **(D)** AMPK induces HMGCR downregulation, resulting in decreased cholesterol synthesis. Moreover, **(E)** AMPK upregulates ABCA1 and its related targets, thereby increasing cholesterol efflux, and simultaneously **(F)** upregulates CPT1A, which further enhances FAO and promotes lipolysis. Collectively, these effects lead to reduced lipid accumulation in the endothelial cells and blood vessels, thereby mitigating ASO. ox-LDL, oxidized LDL; SREBPs, sterol regulatory element-binding proteins; AMPK, AMP-activated protein kinase; ACC, acetyl-CoA carboxylase; FAO, fatty acid oxidation; ABC, ATP-binding cassette; mTOR, mammalian target of rapamycin; FAT, fatty acid transport; CPT - 1, carnitine palmitoyltransferase-1; FABP4, fatty acid binding protein 4; SR-BI, scavenger receptor class B type I; GPx, glutathione peroxidase; SOD, superoxide dismutase; FASN, fatty acid synthase; PI3K, phosphatidylinositol-3-kinase; PD - 1, programmed cell death protein -1; CTLA - 4, cytotoxic T lymphocyte associated protein 4; Tim-3, T cell immunoglobulin and mucin-domain containing-3; HMGCR, 3-hydroxy-3-methylglutaryl-coenzyme A reductase; CEH, cholesterol ester hydrolase; PPAR, peroxisome proliferator–activated receptors.

Immune checkpoints affect lipid metabolism by regulating the metabolic pathways of immune cells (47). The activation, differentiation, and functions of immune cells are critically dependent on the dynamic homeostasis of lipid metabolism (134). In immune cells, Tim-3, PD - 1, and CTLA - 4 collaboratively inhibit the glycolytic pathway and enhance PPAR/AMPK-dependent FAO, and also reduce lipid biosynthesis by suppressing PI3K/Akt/mTORC1 signaling (135–137). These regulations may drive T cells and macrophages to favor lipids as their energy source, thereby lowering lipid accumulation within arterial plaques. For example, Tim-3 inhibition has been shown to increase plaque area in mice fed with high-fat diet (123). Here, we discussed how immune checkpoints regulate lipid metabolism and affect ASO from specific immune checkpoint molecules (**Table 1**, **Figure 2**).

**Table 1:** Functional interaction of the major immune checkpoints and lipid metabolism during arteriosclerosis obliterans.

Immune checkpoints and their ligands	Roles in lipid metabolic effects and ASO	Author
PD-1/PD-L1	Decrease glycolysis and amino acid metabolism in CD4^+^T cells while significantly enhance FAO	Patsoukis et al. (135)
	Activates the PI3K/Akt pathway, downregulates FASN, and inhibits fatty acid biosynthesis	Soltani et al. (137)
	Inhibit glycolysis and phagocytic activity of macrophages, making macrophages to preferentially use lipids as an energy source	Gordon et al. (138)
	Reduce T cell-mediated inflammation and decrease plaque area and increases atheroprotective circulating ox-LDL-specific IgM levels	Grievink et al. (118)
Tim-3/Galectin-9	Suppresses glycolysis in T cells, thereby driving T cells to rely more on FAO for energy production	Lee et al. (143)
	Inhibit M1 polarization and downregulate CD36 and SR-A expression of macrophages, thereby reducing the uptake of ox-LDL	Yu et al. (144)
	Plays a negative regulatory role in atherosclerosis, and decrease plaque formation.	Foks et al. (123)
	Regulates the inflammatory response in ASO by inhibiting NF-κB activation	Lian et al. (122)
CTLA-4/CD80/86	Inhibits T cell glycolysis	Patsoukis et al. (135)
	Reduce enzymes related to fatty acid synthesis such as FASN	Zhang et al. (140)
	Reduce genes associated with cholesterol metabolism such as HMGCR	Pokhrel et al. (141)
	Inhibition of CTLA - 4 signaling accelerates atherosclerosis development by NF-κB-mediated Th1-biased immune response	Zhao et al. (114)
	Reduce atherosclerotic lesion formation and plaque accumulation of macrophage and T cells	Matsumoto et al. (113)

### 4.1 PD - 1/PD-L1

PD-1 is an important regulator of the immune system and essential for regulating lipid metabolism. Upon PD-L1 binding to PD - 1 and subsequent activation, T cells are unable to carry out glycolysis and amino acid metabolism normally, yet endogenous FAO is enhanced as PD - 1 upregulates the lipases CPT1A and ATGL, thereby promoting endogenous lipolysis and FAO (135). Moreover, PD - 1 exerts metabolic regulatory effects on macrophages. Recent studies have shown that PD - 1 signaling markedly inhibits glycolysis and phagocytic activity in tumor-associated macrophages (138). These metabolic changes may cause macrophages to preferentially use lipids as an energy source, thereby reducing lipid accumulation in arterial plaques and slowing the progression of atherosclerosis. However, the precise mechanism through which PD - 1 regulates lipid metabolism requires further investigation. We hypothesized that a similar mechanism might operate in ASO to reduce plaque formation. Additionally, patients treated with PD - 1/PD-L1 inhibitors for tumors exhibit an increased risk of cardiovascular events, including the aggravation of atherosclerotic occlusive disease (139). Thus, the PD - 1/PD-L1 axis offers a new perspective for ASO treatment. Furthermore, caution should be exercised when using PD - 1 inhibitors in cancer patients with coexisting ASO.

### 4.2 CTLA - 4

Studies have indicated that CTLA - 4 inhibits glucose uptake in Treg cells; however, unlike PD - 1, it does not significantly enhance FAO in T cells (135). In other words, CTLA - 4 primarily maintains T cells in a metabolically suppressed or homeostatic state rather than actively triggering lipid metabolic pathways. Moreover, in a tumor microenvironment, the CTLA - 4 signaling pathway can affect blood lipid concentrations by regulating enzymes related to fatty acid synthesis (such as FASN) and genes associated with cholesterol metabolism such as *HMGCR (* 140, 141). In mice subjected to antibody-mediated CTLA - 4 blockade, cholesterol synthesis and LDL uptake significantly increased, exacerbating atherosclerotic lesions (142). Although multiple lines of evidence have shown that CTLA - 4 is closely associated with lipid metabolism and plays a role in slowing atherosclerosis, further studies are needed to investigate how CTLA - 4 maintains T cell homeostasis to reduce plaque formation during ASO.

### 4.3 Tim-3

Experiments showed that in Tim-3-overexpressing Jurkat T cell lines, glucose uptake, lactate production, and glucose transporter-1 concentrations were downregulated, whereas Tim-3 knockout exhibited opposite effects (143). This indicates that Tim-3 signaling suppresses glycolysis in T cells, potentially driving T cells to rely more on FAO for energy production. Moreover, Tim-3 also influences lipid metabolism in macrophages. CD36 and SR-A are primarily responsible for the uptake of lipoprotein-derived cholesterol by macrophages, and are predominantly expressed in M1 macrophages (144). Tim-3 may inhibit M1 polarization, thereby downregulating the expression of CD36 and SRA. Thus, Tim-3 may reduce ox-LDL uptake, decrease foam cell formation, and slow ASO progression. However, studies also found that in human monocyte-derived macrophages, Tim-3 overexpression suppresses miR-155-induced cholesterol ester hydrolase (CEH) expression. Furthermore, miR-155 normally promotes macrophage cholesterol efflux and reduces cholesterol ester accumulation by upregulating CEH expression, thereby inhibiting foam cell formation and atherosclerosis development (145). This implies that Tim-3 accelerates atherosclerosis progression by inhibiting the miR-155-CEH axis. Thus, in-depth studies of the regulatory mechanisms of Tim-3 may provide new directions and targets for ASO treatment.

## 5 Conclusion and prospects

ASO is a complex chronic vascular disease characterized by disruption of lipid metabolism and chronic inflammation. Lipid accumulation, foam cell formation, and inflammatory responses are critical in ASO development, while immune checkpoints, such as PD - 1, CTLA - 4, and Tim-3, serve as key regulatory elements in the interplay between lipid metabolism and immune activity. These immune checkpoints may affect the metabolic and inflammatory environments of ASOs, thereby influencing lipid uptake, FAO, cholesterol efflux, and macrophage polarization. Therefore, immune checkpoint molecules are potential biomarkers for early ASO diagnosis. Drugs targeting immune checkpoints could be developed to delay ASO progression and prevent postoperative recurrence. Further exploration of the interactions between lipid metabolism regulators (such as AMPK, PPARs, and LXR) and immune checkpoints may reveal novel pathways and potential therapeutic targets for ASO management.

Although immune checkpoint inhibitors can enhance immune responses to tumors by blocking checkpoints and have become a breakthrough in cancer therapy (146), studies on immune checkpoint inhibitors have demonstrated their potential effect on cardiovascular diseases, including heart failure and arteriosclerosis (125, 147). Many studies have reported adverse events related to atherosclerosis caused by immune checkpoint inhibitors (130, 148). Therefore, enhanced monitoring of ASO and cardiovascular risk in patients undergoing immune checkpoint inhibitor therapy is recommended.
